# Oral vaccination as a potential strategy to manage chronic wasting disease in wild cervid populations

**DOI:** 10.3389/fimmu.2023.1156451

**Published:** 2023-04-14

**Authors:** Scott Napper, Hermann M. Schatzl

**Affiliations:** ^1^ Vaccine and Infectious Disease Organization, University of Saskatchewan, Saskatoon, SK, Canada; ^2^ Department of Biochemistry, Microbiology and Immunology, University of Saskatchewan, Saskatoon, SK, Canada; ^3^ Calgary Prion Research Unit, Faculty of Veterinary Medicine, University of Calgary, Calgary, AB, Canada

**Keywords:** chronic wasting disease, oral vaccine, wildlife, prion, cervid

## Abstract

Prion diseases are a novel class of infectious disease based in the misfolding of the cellular prion protein (PrP^C^) into a pathological, self-propagating isoform (PrP^Sc^). These fatal, untreatable neurodegenerative disorders affect a variety of species causing scrapie in sheep and goats, bovine spongiform encephalopathy (BSE) in cattle, chronic wasting disease (CWD) in cervids, and Creutzfeldt-Jacob disease (CJD) in humans. Of the animal prion diseases, CWD is currently regarded as the most significant threat due its ongoing geographical spread, environmental persistence, uptake into plants, unpredictable evolution, and emerging evidence of zoonotic potential. The extensive efforts to manage CWD have been largely ineffective, highlighting the need for new disease management tools, including vaccines. Development of an effective CWD vaccine is challenged by the unique biology of these diseases, including the necessity, and associated dangers, of overcoming immune tolerance, as well the logistical challenges of vaccinating wild animals. Despite these obstacles, there has been encouraging progress towards the identification of safe, protective antigens as well as effective strategies of formulation and delivery that would enable oral delivery to wild cervids. In this review we highlight recent strategies for antigen selection and optimization, as well as considerations of various platforms for oral delivery, that will enable researchers to accelerate the rate at which candidate CWD vaccines are developed and evaluated.

## Prion diseases

Prion diseases are a unique category of infectious disease in which the molecular basis of infectivity resides in the misfolding of a normal cellular protein (PrP^C^) into a pathological, self-propagating conformation (PrP^Sc^) (1). Prion diseases can arise from genetic polymorphisms that predispose PrP to misfold, uptake/ingestion of PrP^Sc^ from the environment or dietary sources, iatrogenic transmission, or sporadic forms that lack a defined cause (2).

### Chronic wasting disease

Chronic wasting disease (CWD) is a prion disease of cervids, including deer, moose, and elk (3). Since its first characterization within captive mule deer of Colorado and Wyoming in 1967, CWD has made steady progression through wild cervid populations of North America (3). As of late 2022, CWD has been detected in free ranging cervids in 30 US states and 4 Canadian provinces (www.usgs.gov). Prevalence of CWD has reached over 40% for free-ranging populations in endemic areas and can be as high as 80–90% in captive populations (4–6). This pattern of emergence and spread suggests CWD is a relatively new disease, likely originating within the last hundred years. Prior to that, there may have been isolated, sporadic cases, but, for undefined reasons, the disease did not become endemically established until more recent times.

While often regarded as a North American problem, CWD has been detected in South Korea as well as three Scandinavian countries (7, 8). While the South Korea cases of CWD appear to be imported from North America, comparative transmission studies indicate sufficient differences to indicate that the European cases likely represent sporadic disease (9–11). This highlights the potential for spontaneous emergence of CWD, as well as new forms of the disease, in previously uncontaminated ecosystems. The extent to which CWD will gain a foothold within these regions has yet to be determined.

Over recent decades CWD has had substantial impact on the health and viability of North American cervid populations (6, 12). Should CWD continue its current trajectory, the anticipated outcomes range from a dramatic reduction in cervid numbers to a complete loss of these species (5, 13). Even the most optimistic of these outlooks is cause for considerable concern. Outside of their intrinsic importance, cervids are critical components of delicate ecosystems; threats to cervid health are certain to have negative consequences on other species as well threatening food security for Indigenous and Arctic populations. There is also tremendous economic activity associated with both wild and farmed cervids. Elk and deer farms, once thriving industries within North America, have suffered greatly since the emergence of CWD (14). The big game hunting industry, valued at over 26 billion dollars in the US in 2016, has also suffered considerable setbacks (6, 12). As damaging as these impacts have been, it is not difficult to envision scenarios, such as the disease spilling over into additional species, that would result in far more dire consequences.

#### Host range of chronic wasting disease

Outside the immediate threat to cervids, the extent to which CWD may threaten other species, including humans, remains a critical question. Fortunately, the transmission of prion diseases across species is restricted, to varying extents, by species barriers. For example, during the BSE crisis of the 1980s, species barriers served to protect millions of people who consumed prion-infected beef, limiting disease transmission to approximately two hundred unfortunate individuals who contracted variant Creutzfeldt-Jacob disease (vCJD), a fatal, untreatable neurodegenerative disorder (15). Species barriers reflect the ability of infecting prions to initiate misfolding of host PrP (16–18). This, in turn, depends on sequence differences between the invading and host PrPs, as well as structural characteristics of the infecting prion amyloid. There is not an established method for predicting the ease of transmission across various species, although different species are known to have unique susceptibilities to prion infection (19).

It is encouraging that infection studies of transgenic mice expressing ovine, bovine, and human PrP indicate minimal transmissibility of CWD (20–23). CWD has, however, been experimentally transmitted to several species, including cattle, pigs, cats, hamsters, and bank voles (24–27). While these infection models often utilize doses and routes of infection that differ from “real world” infection, this nevertheless highlights the theoretical potential for CWD to infect these species. Of which, the spectre of transmission of CWD to cattle is particularly concerning as this could result in a “second-generation” BSE outbreak of similar economic and human health consequences as the first, but with the additional challenge associated with managing an environmental source of infection. There is also considerable concern that the northern migration of CWD could result in transmission to caribou which are an important food source for Northern communities and whose numbers and extensive patterns of migration could provide a mechanism to further accelerate the geographical spread of the disease (28).

There is conflicting evidence on the extent to which CWD represents a threat to human health. Opportunities certainly exist for zoonotic transmission; it is estimated that approximately 10,000 CWD-infected cervids are consumed by humans each year (29). While there isn’t an obvious increase in the rate of human prion disease amongst consumers of cervid meat, this must be balanced with the appreciation that transmission is likely quite inefficient, that the number of people consuming cervid food products is low (at least relative to those consuming beef products during the BSE outbreak), and that rates of occurrence of human prion disease must be evaluated against a baseline of sporadic cases, which occur at a rate of one to two cases per million people annually (30). With that, establishing the zoonotic potential of CWD from epidemiological data may be problematic, particularly if human CWD should manifest with similar symptoms as CJD. Several experimental studies support the zoonotic potential of CWD, including a recent study in which infection of transgenic mice expressing human PrP resulted in atypical disease and fecal prion shedding (31). The efficient *in vitro* conversion of human PrP by CWD prions (32, 33) also supports the zoonotic potential. With respect to the transmissibility of CWD to non-human primates, squirrel monkeys are susceptible to intracerebral and oral infection (34). However, studies in *Cynomolgus* macaques, generally regarded as the most relevant non-human primate model for zoonotic transmission studies, present conflicting results; some efforts indicate an absence of transmission (34–36) while other studies show susceptibility to both oral challenge and intracerebral infection (37).

It is also important not to adopt too reductionist of a perspective on the zoonotic potential of CWD, nor to consider the disease as a static threat. Transmission of CWD to humans through intermediate species, including cattle or pigs, could have the same functional consequences as direct transmission from cervids. Further, the existence of various CWD prion strains, as well as the potential for new strains of novel traits, including species tropisms, also needs to be acknowledged. Such strains could emerge during passage within cervids or through any number of intermediate species. The number and diversity of PrP sequence polymorphisms within species sharing the environment with cervids offers troubling opportunity for the emergence of new strains. Collectively, given the fatal and untreatable nature of prion diseases, coupled with the dynamic nature of the threat, a conservative approach to zoonotic potential of CWD seems justified.

#### Efforts to control chronic wasting disease

Outbreaks of other prion diseases have been successfully managed in the absence of a vaccine. For Kuru, this was achieved through alterations to human behavior with the cessation of cannibalistic funeral rituals (38). For BSE, changes to animal management practises, in particular removal of high-risk materials from animal feed, was sufficient to control the disease (39). Unfortunately, aspects of CWD make its control far more problematic. Firstly, the existence of CWD within wild animals complicates disease surveillance as well as implementation of control measures. Animals infected with CWD shed substantial amounts of prions into the environment *via* their urine, feces, and saliva (40, 41). Once in the environment, these prions display remarkable durability, resulting in long-term contamination of soil and water, which provides additional mechanisms for geographical spread and undermines efforts to protect farmed cervids through tightened biosecurity (42, 43). Finally, that CWD is among the most contagious of the prion diseases further challenges its management (3).

Thus far the efforts by U.S. and Canadian government agencies to manage CWD have fallen short of desired goals. Even within the controlled environment of farmed animals, the efforts to manage CWD through double-fencing, increased restrictions on the transport of animals, decommissioning and depopulation of infected farms, have been insufficient to control the disease at an industry level. Not surprisingly, it has proven even more difficult to manage CWD in wild animal populations. Efforts such as depopulation and selective harvesting of animals have been ineffective in stopping the expansion of CWD throughout North America. There is clear and urgent need for new tools to control CWD. Historically, vaccines have been the most effective method for management of human and animal infectious diseases and there is optimism borne of evidence that the development of an effective prion vaccine, including orally administered vaccines, is achievable.

## Opportunities and challenges for CWD vaccines

Relative to many of the other proteinopathies, the prion diseases are advantaged for vaccine development in that PrP represents a clearly defined, cell-surface accessible, immunotherapeutic target. Further, numerous investigations confirm the ability for antibodies to PrP to inhibit prion propagation *in vitro* as well as for passive and active immunization to inhibit disease progression in animal models. While encouraging, the development of prion vaccines is challenged by unique aspects of prion biology including defining safe and protective antigens, overcoming immunological tolerance, and obtaining a better understanding of the extent, and mechanisms, by which immunotherapy impacts disease initiation and progression. This information is critical to provide rationale criteria for optimizing desired vaccine traits as well as allowing the establishment of realistic benchmarks of vaccine efficacy.

## Components of a CWD vaccine

### Antigen selection

For traditional infectious diseases, the vaccine antigen is represented either by the entirety (killed or attenuated vaccines) or a specific molecular component (subunit vaccines) of the disease-associated microbe. Prion diseases are unique in that the entirety of the infectious threat is represented by a single protein. While this seemingly simplifies antigen selection, there are opportunities to utilize limited segments of the protein to achieve conformation specific (PrP^C^ vs PrP^Sc^) immune responses or to prioritize specific regions of PrP based on anticipated outcomes of safety and/or efficiency.

### PrP^C^ as the immunotherapeutic target

**Figure 1:**
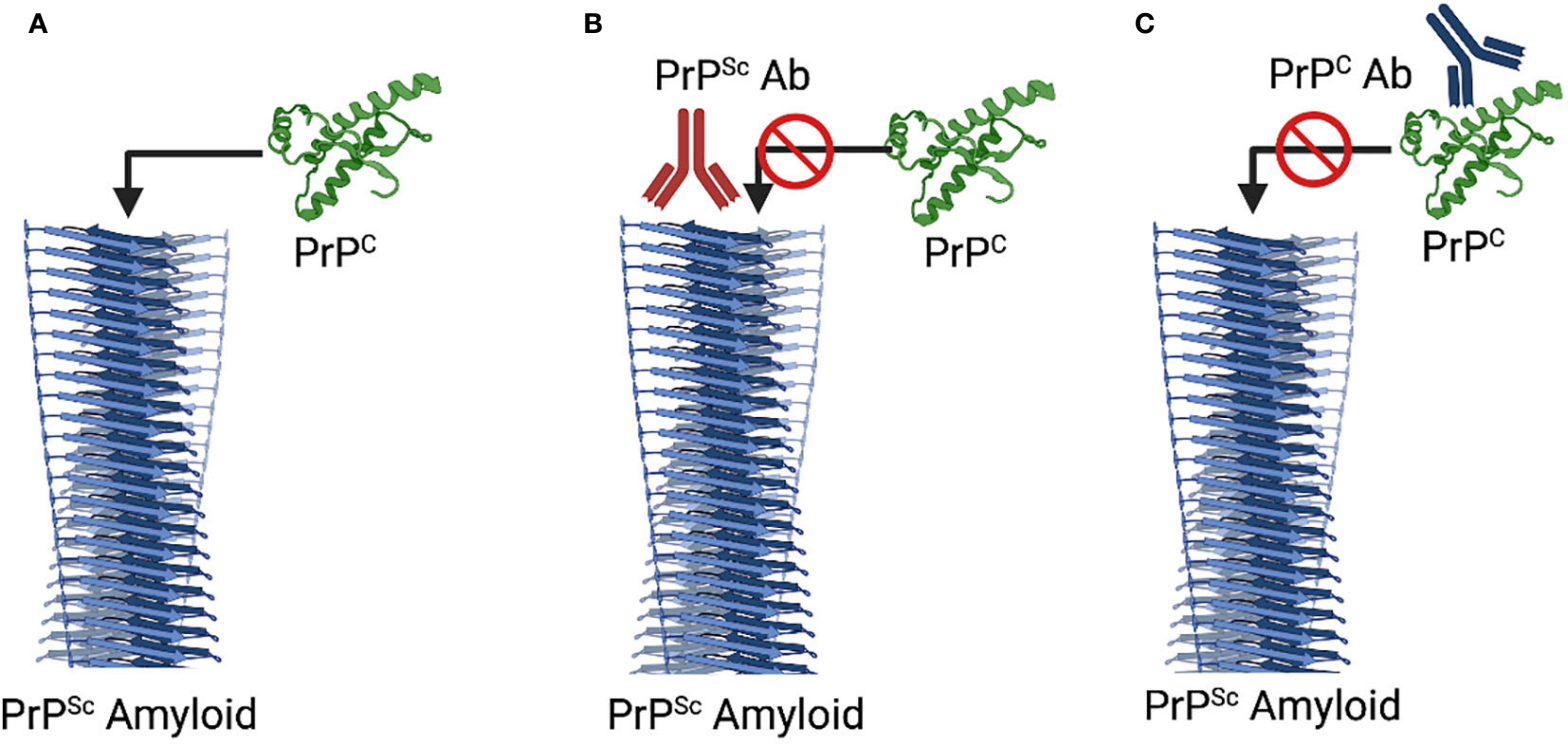
Mechanisms of Immunotherapeutic Intervention. **(A)** Natural Progression. The PrP^Sc^ serves as a template to promote the misfolding and incorporation of PrP^C^. **(B)** PrP^Sc^-Specific Immunotherapy. Antibodies to PrP^Sc^, through disruption of the interaction between PrP^Sc^ and PrP^C^ block induced misfolding of PrP^C^. **(C)** PrP^C^-Specific Immunotherapy. Antibodies to PrP^C^ can block the interaction with PrP^Sc^ as well as depleting PrP^C^. Notably the two mechanisms are not in exclusion of each other such that additive or synergist benefit could occur through dual targeting of each isoform. Diagram created with Biorender.

Given the opportunity to target PrP^C^ or PrP^Sc^, it may seem counterintuitive to prioritize the healthy conformation. There are, however, strong rationalizations for this approach. It is well established that PrP^C^ is essential for prion propagation; prion disease progression cannot proceed in the absence of the PrP^C^ substrate. This is most conclusively demonstrated by the fact that PrP-/- animals completely resist prion infection (44). Efforts to develop vaccines that induce PrP^C^ reactive antibodies look to achieve the same functional outcome through either immunological depletion of PrP^C^ and/or blocking its ability to serve as a substrate for conversion into PrP^Sc^ (45, 46) (**Figure 1**). Antibodies to PrP^C^ block the generation of PrP^Sc^ *in vitro* and extensive investigations have demonstrated that vaccines which induce PrP^C^ reactive antibodies can delay, to varying degrees, the onset of prion disease symptoms (46–49).

Safety is a pivotal consideration during development of any vaccine; this takes on even greater importance for diseases caused by self-proteins. The strategy of deliberately targeting of PrP^C^, which is available for antibody binding in otherwise healthy animals, has raised apprehensions of the safety of this immunological approach. Those concerns are supported by early evidence which seemed to indicate the potential for PrP^C^-reactive antibodies to have pathological consequences. PrP^C^ reactive antibodies, as well as their Fab fragments, were found to induce neuronal apoptosis in the brain (50, 51), although more recent investigations challenge this result (52). Within experimental systems, PrP^C^ reactive antibodies can activate inappropriate cell signal events (53, 54), superoxide mediated cytotoxicity (55), and stimulation of suppressor T-cell lymphocytes (56), although the significance of these responses is unclear.

Reassuringly, there is an absence of reported pathologies from numerous vaccine trials, employing a variety antigens and strategies of formulation and delivery to achieve PrP^C^ reactive immune responses. While this helps to alleviate some of the concerns of targeting PrP^C^, it is important to note that the priority of those investigations was to quantify vaccine efficacy, not safety, and it is possible that subtle vaccine-associated pathologies may have escaped the attention of the researchers. It would also be premature to assume the safety of all PrP^C^-associated vaccines as antibodies with reactivities to different regions of PrP have unique potentials for pathology; antibodies to the octarepeat are well tolerated while those against the folded globular domain are implicated in neurotoxicity (55). Another important consideration is whether the binding of antibodies to various regions are associated with a gain, or loss, of function. Loss of PrP^C^ function seems well tolerated, as evidenced by the absence of any profound phenotypic consequences with genetic ablation of PrP (44). Gain-of-function changes would be more difficult to anticipate in terms of their occurrence and consequences. Thus, while the current evidence suggests the safety and effectiveness of targeting PrP^C^, it is important to evaluate any candidate CWD vaccines on a case-by-case basis. Collectively, however, is generally accepted that PrP^C^ is a viable antigen for prion vaccines, with demonstrated potentially for efficacy in inhibiting disease progression in the absence of adverse side effects.

### PrP^Sc^ as the immunotherapeutic target

Strategies to develop vaccines that restrict the induced immune responses to PrP^Sc^ are motivated by considerations of safety and efficacy. From the perspective of safety, restricting immune responses to the misfolded conformation may mitigate concerns over the safety of induction of immune responses to a widely expressed self-protein; concerns which, whether experimentally demonstrated or merely perceived, could impact vaccine approval, licensure, and utilization. For efficacy, prioritizing the misfolded species could focus the immune response to the most pressing threat while sparing the function of the healthy form of the protein (**Figure 1**). While conceptually appealing, conformation-specific immunotherapy depends on identification of epitopes, termed disease specific epitopes (DSEs), that are specifically exposed for antibody binding in the misfolded state.

### Disease specific epitopes

Efforts to identify regions of PrP exposed for antibody binding in the misfolded protein are complicated by the tendency of PrP^Sc^ to form insoluble aggregates which are unsuitable for most biophysical techniques. Instead, the initial PrP DSEs were identified through lower resolution biophysical techniques, rationale deduction, and bioinformatic approaches. The first DSE emerged from a biophysical investigation which indicated that misfolding of PrP^C^ to PrP^Sc^ resulted in the surface exposure of tyrosine residues which was further determined to correspond a YYR motif of beta-strand 2 (57, 58). Soon after, a second DSE, corresponding to a YML sequence of the opposing beta-strand was hypothesized, and confirmed, to undergo similar repositioning to surface exposure with misfolding (59). A third DSE, corresponding to the loop region between beta-strand 2 and alpha-helix 2, was identified through a bioinformatic approaches that anticipate protein regions with the greatest propensity to unfold (60). This third DSE was designed Rigid Loop (RL) to reflect the unusual rigidity of this region in cervid PrP (61). Notably, high resolution structures of prion amyloids have recently been determined through cryo-electron microscopy, which should enable the identification of additional DSEs (62, 63).

Vaccines to each of the three PrP DSEs have been shown to induce antibodies that can discriminate PrP^C^ and PrP^Sc^ with specific reactivity to the pathological conformation (58, 64). This specificity is maintained in univalent and multivalent vaccine formats (64). Antibodies to each DSEs have also been confirmed in *in vitro* assays to neutralize PrP^Sc^ (65). Of the DSEs, protective efficacy has only been evaluated for a parenterally administered, univalent vaccine based on the YYR DSE. The results of those trials, performed in two different large animal models, revealed conflicting results; the vaccine delayed disease onset in a sheep challenge model (66) but accelerated disease in elk exposed to environmental prions (67). While each model utilized oral routes of infection, the sheep were infected once with a large challenge dose while the elk, housed in a prion-infected environment, were subjected to prolonged, low-level exposure prions. It is not clear whether these different outcomes reflect differences in the species or the challenge models.

Others have developed PrP^Sc^ specific vaccines through the design of recombinant antigens in which discontinuous, surface-exposed residues in PrP^Sc^ are presented in a molecular scaffold designed to mimic a proposed 4-rung beta solenoid fold of PrP^Sc^ (68). While recent determinations of the structure of the prion amyloid through cryo-electron microscopy challenge the beta solenoid model of PrP^Sc^ (62, 63), a vaccine based on this antigen, termed VPrP^Sc^, induced PrP^Sc^-reactive antibodies and resulted in a dramatic delay in the onset of symptoms in a transgenic mouse model of a genetic human prion disease (69).

### Potential dangers of PrP^Sc^ reactivity

Targeting PrP^Sc^, whose presence is unique to prion infection, seems a rational approach to mitigate safety concerns associated with induction of auto-reactive antibodies. This strategy, however, merely shifts, rather than alleviates, the safety concerns. Immunotherapy based on targeting of PrP^Sc^ gives rise to new apprehensions that these antibodies could function as templates, or chaperones, to promote formation of PrP^Sc^. Antibody induced misfolding of PrP^C^ has the theoretical potential to initiate prion disease in otherwise healthy subjects. Thus far, these hypothetical concerns are not supported by experimental evidence. Antibodies to the YYR DSE did enhance the presentation of these regions but failed to generate PrP^Sc^ (57). Similarly, prolonged incubation of brain homogenates with polyclonal antibodies to the three DSEs failed to generate PrP^Sc^ (64, 66). Finally, induction of high titre PrP^Sc^-specific antibodies in prion-disease sensitized transgenic mice did not result in clinical nor biochemical indications of prion disease after eight months (70).

While the inability of PrP^Sc^ reactive antibodies to promote misfolding of wildtype PrP is reassuring, there may be elevated opportunities for antibody-induced misfolding with naturally occurring PrP polymorphisms associated with genetic prion disease. In nanopore and immunoprecipitation experiments, PrP^Sc^-specific antibodies bound to a PrP variant associated with early onset familial dementia, indicating the occurrence, and recognition, of conformational differences and/or partially unfolded species resulting from this mutation (71). Although prolonged *in vitro* incubation of the PrP^Sc^-specific antibodies with the misfolding prone PrP^C^ did not generate PrP^Sc^, this nevertheless raises concern of this strategy of vaccination in outbred populations with a range of PrP polymorphisms. Thus, like the situation with the PrP^C^-specific vaccines, the safety of PrP^Sc^-specific vaccines will need to be evaluated on a case-by-case basis with appropriate consideration of naturally occurring PrP polymorphisms.

Collectively, it is generally accepted that PrP^Sc^ is also a viable target for prion vaccines, with demonstrated efficacy in inhibiting disease progression in the absence of adverse side effects. Importantly, antigen selection for a prion vaccine may not need to represent an either/or situation of PrP^C^ versus PrP^Sc^. As each target offers distinct, and potentially complimentary benefits to inhibit disease progression there could be value in the development of vaccines which induce antibodies against both conformations. This outcome which could be investigated through either a single antigen or a multivalent approach that combines top candidate PrP^C^ and PrP^Sc^ reactive antigens.

### Antigen optimization (overcoming self tolerance)

Independent of the desired specificity, overcoming immunological tolerance is a shared challenge to the development of any prion vaccine. Immune tolerance refers to the unresponsiveness of the immune system to self-molecules due to developmental depletion of T and B lymphocytes with reactivities to self-antigens. Immune tolerance serves to prevent autoimmune disorders but also opposes efforts to develop vaccines against self-proteins. PrP^C^ falls within the jurisdiction of immune tolerance, and, as the conversion to PrP^Sc^ does not involve alteration to the polypeptide sequence, immune privilege also extends to the pathological isoform. Consequently, most prion infections progress to their fatal outcomes in the absence of an induced immune response (72–76). Thus, the unique biology of prion infection offers sanctuary from immune activation, enabling unfettered disease progression, and challenging the development of vaccines; overcoming immune tolerance is a central obstacle to the development of prion vaccines (77).

As immune tolerance is based on host PrP, one strategy to overcome self-tolerance is to utilize PrP from heterologous species as vaccine antigens; that species-specific variations of PrP sequence can provide versions of the protein that are recognized as foreign by the immune system. For example, while mouse PrP fails to induce immune responses in BALB/c mice (78), bovine and sheep PrP were highly immunogenic (79). A stipulation to this approach is ensuring that the antibodies induced to the heterologous PrP have sufficient cross-reactivity to enable binding of the infecting and/or host PrP. There are examples in which antibodies to heterologous PrP antigens are unable to bind PrP^C^ or PrP^Sc^ (80). Alternatively, presentation of PrP as aggregation-prone recombinant dimers can also overcome immune tolerance, even to homologous sequences (45, 46). Finally, alternative strategies of antigen formulation and delivery can overcome self-tolerance; presentation of PrP in the context of Dynabeads (81) or polylactide-coglycolide nanospheres (48) have enabled creation of immunogenic vaccines.

The obstacle of self-tolerance is further complicated for peptide-based vaccines, as these minimal antigens are often weakly immunogenicity. Early efforts to translate the DSE sequences into vaccines faced considerable challenges; a vaccine based on the YYR DSE induced only weak IgM responses, even when conjugated to an immunogenic carrier and formulated with harsh adjuvants (57). One effective strategy to improve the immunogenicity of these peptides is to expand their lengths through the inclusion of naturally occurring residues flanking the region of interest. In performing these expansions, it is critical to ensure that PrP^Sc^ specificity is maintained; that increased immunogenicity is not at the expense of PrP^Sc^ specificity. The direction and extent of expansion of these core sequences can be performed through *in silico* analysis to anticipate immunogenicity based on the inclusion of endogenous B-cell epitopes (82). Through optimization of core sequences, as well as presentation of these optimized sequences on suitable carrier proteins, the three DSEs were translated into vaccines that exhibit strong immunogenicity while maintaining PrP^Sc^ specificity (64).

Rationale selection and optimization of peptide antigens is critical, but often insufficient, to overcome immune tolerance. To elicit the T-cell help required for strong immune humoral responses peptide antigens must usually be presented in the context of immunogenic carrier proteins. A variety of carrier proteins have been investigated including Leuktoxin of *Mannheimia haemolytica* (58), rabies glycoprotein G (83), blue carrier protein (84), cholera toxin (85), heat-labile enterotoxin B subunit (86) and heat shock proteins (87). Certain carriers, such as cholera toxin and *Escherichia coli* heat-labile enterotoxin, are better suited for mucosal vaccines (88) while others, like rabies glycoprotein, are of particular interest for their ability to induce strong, sustained immune responses including within the context of oral vaccines (89, 90).

## Biological vectors for an oral CWD vaccine

Many of the initial efforts to develop CWD vaccines involved parenteral administration, this allowed researchers to prioritize the identification of protective antigens without the additional challenges associated with oral vaccines. While injected vaccines could find application for farmed cervids, there is recognition of the eventual necessity for orally administered vaccines. Outside of the obvious practical perspectives of vaccinating wild animals, there is also emerging recognition that protection against CWD infection, which occurs through oral routes, may depend on the induction of strong mucosal responses.

Several successful examples of oral vaccines for wildlife offer assurance of the viability of this approach and may provide framework for design of oral CWD vaccines. Most notably, oral rabies vaccines have been incredibly successful in management of that disease (91). As the protective antigen of those vaccines, glycoprotein G, is also an effective carrier for PrP antigens, it may be possible to transform established oral vaccines for rabies into CWD vaccines through the simple inclusion of the additional PrP epitopes. That effective oral rabies vaccines, utilizing different biological vectors to deliver the glycoprotein G gene, support the versatility of this approach for adaptation to cervids.

### Adenovirus vectors

One of the commercialized oral rabies vaccines, OnRab, utilizes a human adenovirus platform to deliver genetic material corresponding to the rabies glycoprotein G protein (89). This system demonstrates considerable potential as an oral vaccine platform for CWD as it possesses a broad species and tissue tropism, induces systemic and humoral immunity, and can be orally dosed (92, 93). A candidate oral CWD vaccine was constructed using a replication incompetent human adenovirus encoding the truncated rabies glycoprotein G with an expanded C-terminal region to represent a series of tandem repeats of the RL DSE. Following oral administration to white-tailed deer this vaccine induced PrP^Sc^-specific systemic and mucosal immune responses after two immunizations, confirming the ability of the vector to infect cells of the cervid gastro-intestinal tract (83). There were no indications of adverse health effects and shedding of the vector was limited to a brief period following administration (83). There is opportunity to build on these highly promising results by using replication-competent virus, which is anticipated to achieve in greater levels of antigen expression with superior immune responses (94).

### Vaccinia virus

A second licenced oral rabies vaccine, RABORAL V-RG, utilizes an attenuated recombinant vaccinia virus vector engineered to express the rabies virus glycoprotein G. This vaccine has proven highly effective in controlling rabies without any reports of adverse reactions in wildlife or domestic animals (90). Vaccinia virus (VV), most famous for serving as the foundation as the smallpox vaccine, has shown considerable potential for the development of vaccines for other infectious diseases due to its large genome which can accommodate large inserts (10-15kb) of foreign genes, established safety profile, stable antigen expression, and ease of storage (95).

### Lambda phage

Bacteriophage are structurally stable, amenable to genetic manipulation, strongly immunogenic, and, as they are omnipresent within the mammalian digestive tract and replicate exclusively within bacteria, generally regarded as safe to eukaryotes (96). These traits are all consistent with an oral delivery platform. This was investigated through presentation of the three PrP DSEs as recombinant fusions of the capsid head protein D of lambda phage. These modified phage particles were taken up by the Peyer’s patches of the small intestine of calves resulting in the induction of strong mucosal (IgA) responses to the peptide epitopes (97).

### Bacterial delivery

Some of the earliest efforts to develop oral prion vaccines utilized attenuated strains of *Salmonella enterica* expressing tandem copies of PrP (98). These attenuated strains reached the gut lymphoid follicles of deer, enabling antigen delivery and induction of immune responses in the absence of any pathologies (99). These oral vaccines resulted in a significant delay in the onset of CWD in white-tailed deer with one of the animals, who demonstrated particularly high anti-PrP titres in both saliva (IgA) and blood (IgG), remaining symptom free after 3 years (100). While the extensive vaccination protocol, involving eight immunizations, would limit the real-world potential of these results, these efforts nevertheless highlight the potential for oral vaccines to serve as a valuable tool for control of CWD.

### Systemic vs mucosal responses

The route of vaccine delivery impacts the nature of the induced immune response. Parenterally administered vaccines tend to favor peripheral humoral responses (IgG) with muted responses of mucosal antibodies (IgA) while mucosal administration favors a more balanced IgG/IgA response (101). This holds true for prion vaccines; parenteral administration of a DSE-based prion vaccine resulted in IgG responses which were an order of magnitude higher than the IgA antibodies while the same epitope, delivered through oral administration with a viral vector, induced a balanced serum IgG to fecal IgA responses (58, 83). A similarly balanced epitope-specific IgG and IgA responses were achieved with mucosal delivery of prion vaccines through bacterial vectors (98–100) as well as carrier proteins specialized for mucosal delivery (86, 88).

Control of CWD within wild animal populations necessitates the use of oral vaccines. With that, it is important to consider how this route of delivery could impact disease control at the level of individual animals as well as the overall population. Orally transmitted prion diseases, including CWD, occur in three stages; uptake at mucosal surfaces, peripheral amplification, and transmission to the CNS (102, 103). It is necessary to contemplate how each of these stages offers unique potentials and challenges for immunotherapeutic intervention as relating to different routes of vaccine administration.

**Figure 2:**
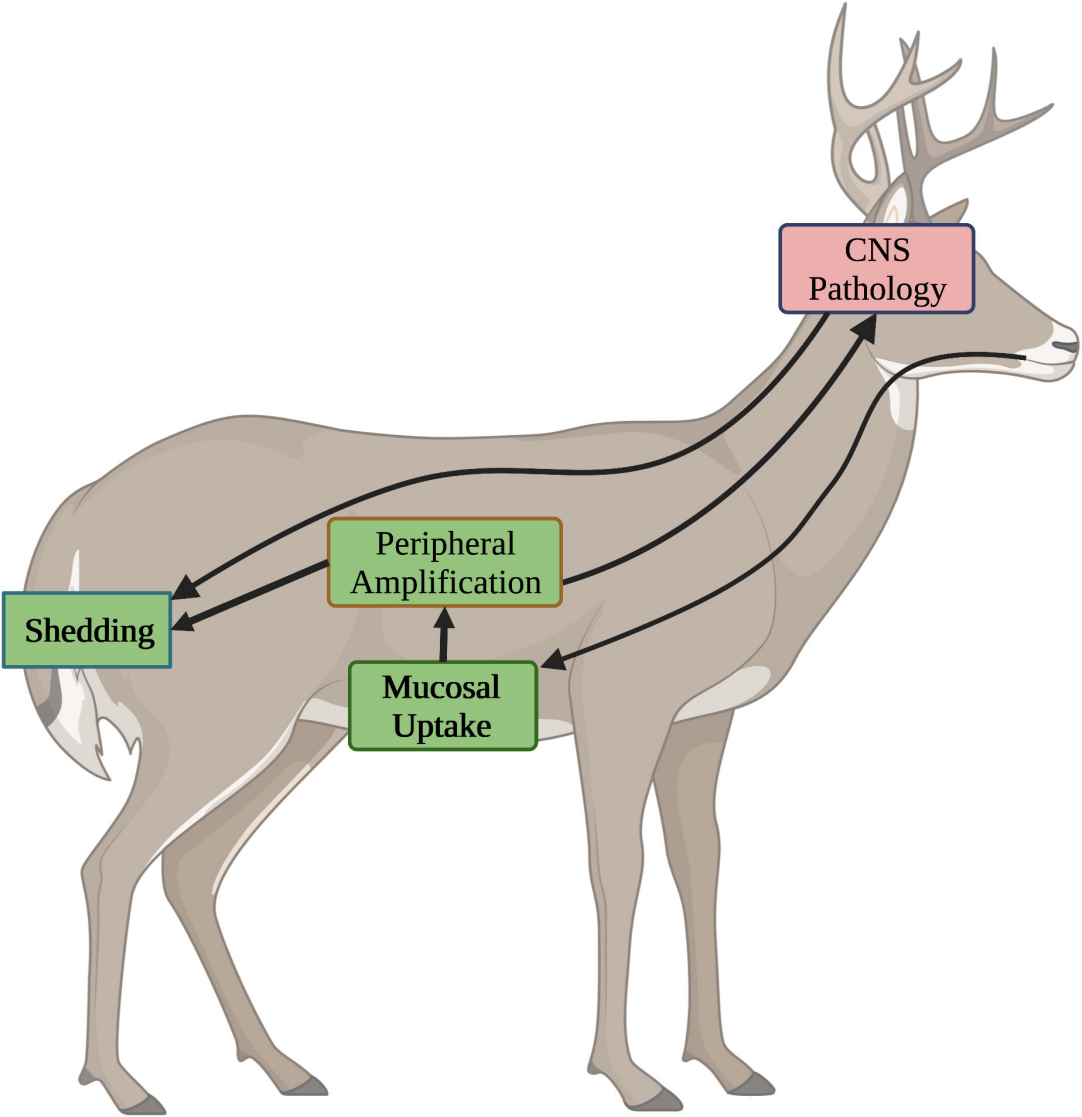
Stages of CWD and Opportunities for Immunotherapeutic Intervention. CWD progresses through four different stages, each of which presents distinct opportunities and challenges for immunotherapeutic intervention. Stages where oral vaccines are anticipated to have positive impact are highlighted in green. Stages which are unlikely to impacted by oral vaccines are shown in red. 1) Mucosal Uptake. Following oral ingestion, environmental prions are taken up through mucosal surfaces. Induction of IgA antibodies through oral vaccines offer the best chance to block uptake. 2) Peripheral Amplification. Following uptake, prions undergo a stage of peripheral amplification. Induction of IgG antibodies, through either oral or injected vaccines can inhibit this process. 3) Shedding. Prions generated in periphery and CNS of the infected host are shed in saliva, urine, and feces. IgG antibodies, induced through injected or oral vaccines, may restrict prion amplification to reduce shedding. 4) CNS Pathology. After peripheral amplification, prions migrate to the CNS where they exert pathological consequences. While the BBB limits access of antibodies to the CNS, IgG antibodies, induced through either oral or injected vaccination, may minimize pathology.

In general, the most effective way to deal with an infectious disease is to prevent it from occurring. This seems particularly true for prion diseases as once infection had initiated, immunotherapy, at least to date, has been limited to slowing, rather than stopping, disease progression. Blocking the uptake, or neutralizing the infectivity, of gut-associated prions could represent an ideal strategy to protect animals from CWD (**Figure 2**). In a best-case scenario, antibodies at the mucosal surface could prevent the uptake of prions from the digestive tract to prevent infection. This is likely dependent on oral vaccinations for induction of mucosal IgA antibodies; the strong peripheral immune responses from parental administration offer greater extent of protection in peripheral rather than oral challenge models (104).

During the second stage of CWD infection, peripheral amplification, the priority shift to slowing disease progression by blocking the ability of PrP^Sc^ to recruit new PrP^C^ into the growing amyloid. This can be achieved by neutralizing the infectivity of PrP^Sc^, depleting PrP^C^, or both. Those mechanisms utilize IgG antibodies as effectors of the response. The consequences of slowing the production of PrP^Sc^ are two-fold. First, this may delay the time frame of which peripheral loads of PrP^Sc^ reach thresholds that promote spillover of the disease to the CNS. As the symptoms of prion disease are associated with CNS pathology, this would serve to prolong the asymptomatic period between infection and onset of symptoms. While this would certainly be valuable for vaccines against human prion diseases, it is less important for controlling CWD within wild populations. Indeed, prolonging the lifespan of an infected animal, enabling greater opportunities for shedding prions into the environment, could be counterproductive. Strong peripheral responses, which inhibit the progression of PrP^Sc^, may serve to reduce the infectious load generated within an animal. This, in turn, would reduce the amount of infectious material released to the environment which, in time, could serve to slow disease progression at the population level (**Figure 2**). While strong peripheral responses are likely best achieved through parenteral administration, oral vaccines may also induce high enough levels of IgG antibodies to reduce the amount of PrP^Sc^ generated, and shed, by infected animals.

The final stage of CWD occurs when the infectious agent reaches the CNS. Here the options for immunotherapy are limited by the impermeability of the blood brain barrier (BBB) to antibodies (105, 106). Concentrations of IgG antibodies in the CNS are typically two to three orders of magnitude lower than in serum (107). This trend has also been observed for prion vaccines (58). With this, neither orally nor parenterally administered vaccines are likely to have much impact on disease progression once the prions have reached the CNS (**Figure 2**). Although reducing peripheral amplification may delay the time required to reach thresholds that favor spillover into the CNS. Prions generated in the CNS will contribute to environmental contamination through retrograde transport to the periphery with subsequent shedding and/or through environmental contamination *via* the animal carcass, including the brain. The minimal ability to slow disease progression in the brain and prolong the lifespan of the infected animal may benefit disease control at a population level by minimizing the duration of time for which infected animals generate and shed prions. As the priority of the CWD vaccines is to protect populations, rather than individuals, the relative inability of orally or parenterally administered vaccines to impact prion disease progression in the CNS may be an acceptable, and even desirable, trait.

Collectively, induction of systemic and mucosal immune responses may be beneficial for an effective vaccine for CWD. When dealing with oral models of prion infection, the greatest extents of protection correlated with high titres of both IgG and IgA antibodies, as compared to either high titres of either IgG or IgA alone (99).

### Prospective impacts of vaccines for control of CWD

Considering the challenges associated with development of effective prion vaccines, it is probably overly optimistic to hope for vaccine that achieves absolute, sterilizing protection. Fortunately, while this extent of protection would obviously be highly desirable, it is not prerequisite for the vaccines to be an asset in control of the disease. Immunization of wildlife could contribute to disease management on two fronts.

Firstly, by diminishing the quantities of prions generated within an infected animal, and subsequently released into the environment, it may be possible to slow, and even reverse, the trend towards an increasing environmental burden of prions which should, in time, be reflected in fewer new cases. That would further serve to further reduce the extent of environmental contamination. Given the durability of prions within the environment and the slow progression of disease within individual animals, this will be a prolonged process. Parallel efforts to decontaminate environments and minimize new infections would serve to complement and enhance this contribution of vaccines to control of CWD.

A second mechanism by which oral CWD vaccines could contribute to control of CWD is through containment of the disease to endemic areas. Given the gradual, predictable, patterns of migration of the disease, coupled with knowledge of the location of populations of vulnerable populations, like the Northern caribou, it may be possible to use a ring vaccination approach to stop, or at least limit, further spread.

The feasibility for strategic placement of oral vaccines, with respect to the timing and geography of vaccine dispersal, to control infectious diseases within wildlife is well supported by the examples of various oral vaccines that have been successfully employed for the control of rabies. Notably, many of the oral vaccines under development for CWD utilize similar biological vectors as the oral rabies vaccines and would have similar traits in terms of cost and environmental durability.

## Conclusions

After decades of research, effective vaccines for the prion diseases, as well as the conceptually related prion-like diseases, such as Alzheimer’s and Parkinson’s disease, remain elusive. While this highlights the magnitude of this challenge, this is an active branch of vaccinology that continues to advance and evolve, offering hope for critical breakthroughs. As these proteinopathies share a common mechanism by which a misfolded self-protein serves as a self-propagating catalyst for additional misfolding events, the lessons learned within vaccine development efforts of each disease, in terms of identification of protect antigens and strategies of vaccine formulation and delivery, may serve common benefit. An important distinction, however, is that the criteria of an “effective” vaccines for human proteinopathies would likely be quite distinct traits than that of a vaccine for control of CWD in wildlife. Within human disease, vaccines that prolong lifespan and minimize disease symptoms would be celebrated achievements. For CWD, however, a vaccine that prolonged the duration of which an infected animal could generate and release prions into the environment would be inconsistent with the goals of disease management, that the priority of CWD vaccines is to save populations, rather than individual animals.

Recent progress in the identification of protective antigens, strategies to overcome immune tolerance, and efforts to translate these approaches into oral vaccines gives hope for the development of oral CWD vaccines. It is critical, however, not to underestimate the challenges presented by CWD, including occurrence in wildlife species, widespread geographic occurrence, environmental persistence, unique molecular mechanisms, and the dynamic nature of the threat (3, 108, 109). Any expectations of the extent and time frames in which the trajectory of the disease can be impacted with a vaccine must be balanced against the magnitude of these obstacles. Indeed, the challenges of CWD are likely too numerous and diverse to hope that any single disease control measure can function as a complete solution. More realistically, an oral CWD vaccine could contribute as a valuable component of a multi-pronged approach that could include strategic culling, utilizing genetic resistance, and decontamination of environmental prions.

## Author contributions

SN and HS co-developed and co-wrote this manuscript. Both authors contributed to the article and approved the submitted version.
